# Low-cost PM_2.5_ sensors can help identify driving factors of poor air quality and benefit communities

**DOI:** 10.1016/j.heliyon.2023.e19876

**Published:** 2023-09-06

**Authors:** Tim Keyes, Rea Domingo, Samantha Dynowski, Royal Graves, Martha Klein, Melissa Leonard, John Pilgrim, Alison Sanchirico, Kate Trinkaus

**Affiliations:** a Evergreen Business Analytics, LLC, USA; b Sacred Heart University, USA; c Sierra Club Connecticut, USA

**Keywords:** Air quality, Particulate matter, PM_2.5_, Reference grade monitors, Low-cost sensors, Calibration modeling, Attribution modeling, Statistical regression, Public health

## Abstract

Air quality is critical for public health. Residents rely chiefly on government agencies such as the Environmental Protection Agency (EPA) in the United States to establish standards for the measurement of harmful contaminants including ozone, sulfur dioxide, carbon monoxide, volatile organic chemicals (VOCs), and fine particulate matter at or below 2.5 μm. According to the California Air Resources Board [1], “short-term PM_2.5_ exposure (up to 24-h duration) has been associated with premature mortality, increased hospital admissions for heart or lung causes, acute and chronic bronchitis, asthma attacks, emergency room visits, respiratory symptoms, and restricted activity days”. While public agency resources may provide guidance, it is often inadequate relative to the widespread need for effective local measurement and management of air quality risks. To that end, this paper explores the use of low-cost PM_2.5_ sensors for measuring air quality through micro-scale (local) analytical comparisons with reference grade monitors and identification of potential causal factors of elevated sensor readings. We find that a) there is high correlation between the PM_2.5_ measurements of low-cost sensors and reference grade monitors, assessed through calibration models, b) low-cost sensors are more prevalent and provide more frequent measurements, and c) low-cost sensor data enables exploratory and explanatory analytics to identify potential causes of elevated PM_2.5_ readings. This understanding should encourage community scientists to place more low-cost sensors in their neighborhoods, which can empower communities to demand policy changes that are necessary to reduce particle pollution, and provide a basis for subsequent research.

## 1 Introduction

Connecticut has the dirtiest air in New England [1] and the consequences to human health are devastating. Globally, air pollution is responsible for nearly 9 million excess deaths annually [2]; in the United States, over 100,000 deaths are attributable to fine particulate matter air pollution [3]; and nearly 200 people die in Connecticut each year from dirty air [4]. On average, every global citizen loses approximately 2.2 years of life due to particulate pollution, comparable to 1.9 average years of life lost due to smoking [5]. PM_2.5_ air pollution is the single greatest global environmental risk factor causing excess death, mostly by cardiovascular disease [6].

There is abundant literature demonstrating negative health impacts from particulate matter. For some time, we have known that exposure to PM_2.5_ increases the risk of death from five conditions: ischemic heart disease, lower respiratory infections, chronic obstructive pulmonary disease, stroke, and lung cancer [7]. More recent studies conclude that the disease burden from PM_2.5_ is higher than previously estimated and is linked to numerous additional illnesses including chronic kidney disease, dementia, type 2 diabetes, hypertension and pneumonia [8].

The burden of excess death attributable to PM_2.5_ exposure disproportionately afflicts Black individuals and low-income communities, and this disparity is apparent in both urban and rural areas. Nearly all the excess deaths occur at levels below EPA safety standards [[8], [9], [10]].

In 2021, the World Health Organization [11] reduced the level of fine particulate pollution considered safe for humans to breathe from 10 μg/cubic meter to 5 μg/cubic meter. This guidance reflects overwhelming evidence showing harm from PM_2.5_ well below levels approved by federal and state agencies. Under the old standard, only 7% of US residents lived in areas considered hazardous; under the new guideline, 93% of us now live in areas where pollution exceeds safe standards [5].

Because of these health effects of air pollution, this study explores drivers of PM_2.5_ and the importance of locality on PM measurement, which should motivate more analysis and be brought to bear on public policy choices regarding management of PM pollution.

Prior to launching this PM_2.5_ research, a recurring strong ammonia odor was observed in an area near the CPV Towantic Energy Center, an 805-MW fossil gas-fueled combined-cycle electric generating facility operating in the Woodruff Hill Industrial Park in Oxford, Connecticut. Adjacent to the Center is the Oxford “Algonquin” Compressor Station, comprised of 3 compressor units at 37,700 total hp; two “Mars-100” turbine-compressors rated at 15,000 hp each and one “Solar Taurus” 60 turbine-compressor rated at 7700 hp; located about 1.4 km east of the Waterbury-Oxford Airport runway (https://www.industrynet.com/listing/3880818/enbridge-inc-algonquin-gas-pipeline-oxford-compressor-station).

Community Scientists in the area deployed a low-cost sensor to measure PM_2.5_ and sought a fugitive methane study from air quality researchers in Connecticut. Using technology reported in an earlier study [12] the team performed a field investigation of the area in April 2022.

**Fig. 1:**
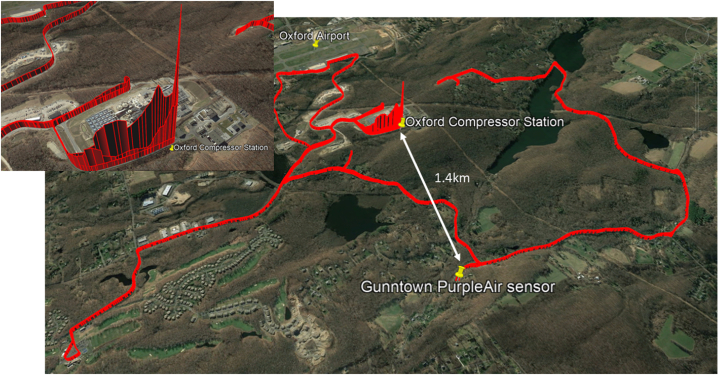
Field study route for measuring fugitive methane; inset: close-up of Energy Center Area (source: Google Earth).

Fig. 1 displays an area map and results of the field study in a data visualization.

From the resulting methane readings, it was clear that the compressor station was producing spikes in methane as compared to ambient levels observed in the surrounding area. The CPV Towantic Energy Center was offline at the time of the fieldwork. Methane observations taken nearer the location of the low-cost sensor later deployed by the Community Scientists produced no noteworthy methane spikes. Subsequent attempts to use airbags to record methane at the sensor location also produced no significant methane readings. To aid in the measurement of air quality in the area surrounding the energy center and compressor station, three PurpleAir (PA) sensors were selected, based on publicly available data, to monitor PM_2.5_, and the present study was launched.

The need for PA sensor *calibration* to reference grade monitor outputs is well documented in the literature [[13], [14], [15], [16], [17]]. Often deployed is a “one-size fits all” approach, meaning a single correction/calibration factor for daily measurements over a wide area, e.g., nation, region, state, etc. based on nearby PA sensors [[13], [14], [15]]. Many calibration studies of low-cost sensors including PA were performed on a *colocation* basis. That is, sensors were placed in close proximity to the reference grade monitor they were intended to mimic [15,18,19]. This was not possible in the micro-region chosen for our analysis over our chosen time-window. Alternatively, we assume the correction/calibration factors determined for each of the three PurpleAir sites would be statistically different, which is supported by the work of Gupta et al. [16], and Self et al. [20].

Beyond determining Local (site-specific) Correction/Calibration Factors (LCFs) by site, we attempt to *attribute* PM_2.5_ variation to local drivers such as temperature, relative humidity, pressure, precipitation, wind speed, wind direction (a categorical variable of compass quadrants with reference or baseline direction being southeast), industrial activity, etc. In peer papers, main causes of PM_2.5_ variation were often assumed *a priori* and defined the setting in which correction/calibration factors were estimated [15,21] while in this study, we are not assuming causality but rather evaluating potential meteorological and industrial correlates as potential driving influences on *hourly* PM_2.5_ variation on a *micro-level*.

Studies have incorporated human, energy-related and/or meteorological factors into their research, but differ in material ways to the present work, chiefly regarding the spatiotemporal data grain or sensor colocation. Geng et al. [22] used *macro-level* exogenous data to quantify the relative influence of eight factors on PM_2.5_-related deaths, including energy policy, economic growth/structure, population growth, and health care. Lim et al. [23] proposed a *macro/regional* spatiotemporal approach to understand PM_2.5_ drivers: population, urban ratio, and vegetation. Brewer et al. [24] found significantly elevated levels of PM_2.5_ and ultrafine particulate concentrations attributed to fossil gas-fired turbines using sophisticated tests conducted on *daily* data at an energy facility in California, U.S.A. Gao et al. [25] quantified the province-specific human health impact of power generation emissions on *annual* mean PM_2.5_ in China. Requia et al. [26] further found *annual regional* PM concentrations vary with meteorological conditions using data available from the EPA and NOAA. Feenstra et al. [27] concluded a decisive influence of Relative Humidity on low-cost sensor bias compared to a *collocated* FEM monitor, and advocated for more refined monitoring on a community or neighborhood scale (a finer spatial grain). Malings et al. [18] created hourly correction equations for *colocated* PA sensors that include Temperature and Relative Humidity as covariates at sites separately impacted by urban activity, industrial activity, traffic emissions and rural activity near Pittsburgh, U.S.A.

As for use of regression to identify potential drivers of PM_2.5_, Russell et al. [28], and Self et al. [20] developed spatiotemporal quantile regressions with meteorological factors on *daily averaged data* and demonstrated significant spatial variation. Our use of logistic regression to evaluate drivers of elevated PM_2.5_ levels among energy and meteorological measurements appears to be novel in the relevant literature.

The present paper expands these efforts by incorporating *hourly* meteorological data, including wind speed, wind direction, and precipitation, while also including *hourly* energy production and *daily* shale (or fossil) gas distribution data in an attempt to evaluate the relative factor influence on elevated levels of locally calibrated PM_2.5_ measured with spatial variation by *non-colocated* sensors.

The goal of this study is two-fold: 1) to evaluate the relationship between PM_2.5_ readings from a state-sponsored Reference Grade Federal Equivalent Method (FEM) Monitor located in Waterbury, CT and readings from low-cost PurpleAir sensors at three nearby locations, in order to investigate if macro-level correction/calibration factors are adequate to develop a localized view on PM_2.5_ variability, and 2) to explore and evaluate, using regression methods, the relationship between calibrated hourly PM_2.5_ readings at each of the three sensor sites, and multivariate meteorological and energy production-related measurements, in order to provide insight into potential driving factors of elevated readings in our chosen micro-area and to provide a blueprint for analysis more broadly across the state, region and beyond.

## 2 Materials and methods

### 2.1 Description of monitor, sensors, and ancillary sources

#### 2.1.1 Waterbury FEM monitor

The Waterbury site uses an FEM Teledyne API T640 monitor for PM_2.5_ mass concentration. The Teledyne API Model T640 with T640X Option is a real-time, continuous PM mass monitor that uses scattered light spectrometry (using 90° white-light scattering with polychromatic LED) for measurement of PM_2.5_, and the T640X Option measures PM_2.5_, PM_10_, and coarse PM. The sample rate is 5 LPM (T640) or 16.67 LPM (T640X) [29].

According to CT DEEP [29], the monitor undergoes automated daily and weekly checks that do not disrupt data collection, but the monitor may be offline for an hour or longer for preventative maintenance or repairs. Full calibrations or audits are performed 2 to 3 times annually and may cause the monitor to be offline for several hours. If unusual data appears during reviews e.g., if a monitor's values are significantly higher or lower than others in the state, CT DEEP may carry out additional quality control checks which may also result in downtime. PM_2.5_ is highly localized so it is unclear why this metric is used as a reason to take a monitor offline for checks.

Hourly PM_2.5_ data (μg m^−3^) from the Waterbury FEM monitor was provided by CT DEEP for the period 1 January 2021 to 1 July 2022. It should be noted that, according to CT DEEP, Eastern Standard Time (EST) is used as the recorded timestamp, and is not adjusted when Daylight Saving Time is used. To facilitate our study, the primary key for all data joins was Universal Time Coordinated (UTC) time (hour). The Waterbury monitor was available 97.5% of the time over the observational period.

#### 2.1.2 PurpleAir sensors (PurpleAir.com)

PA–II–SD air quality sensors use 2 PMSX003 sensors, specifically the Plantower PMS-5003 sensor. These sensors operate using a class 1 laser and a detector plate to measure particulate matter. A fan draws air into the device and through the path of the laser beam, and any passing particles reflect light onto the detector which measures the reflected light pulse. The duration of the pulse indicates the size of the particulate matter, and the number of pulses is used to determine PM concentration. The sensor can differentiate between and measure concentrations for PM_1.0_, PM_2.5_, and PM_10_ for standard indoor (CF = 1) and outdoor PM (CF = ATM). The 2 PMS-5003 sensors measure PM in real time, with each one alternating 5-s readings averaged over 120 s. Hourly PM_2.5_ data (μg m^−3^) were obtained from the PurpleAir website map by location for the Gunntown, Long Meadow, and Lake Zoar sensors.

#### 2.1.3 Ancillary data sources

Table 1 is a summary of data evaluated in this study. As noted, this paper's aim is to explore micro-level calibrations of low-cost sensors to a nearby reference grade monitor, and to further attempt to explain variability in PM_2.5_ by attribution to and determination of the relative importance of potential causal factors both natural and man-made, namely: temperature (temp), relative humidity (RH), time of day, day of week, precipitation, visibility, wind speed, wind direction, energy production, energy distribution, and road/air traffic activity. Insufficient data limited useful analysis for some sources, while other sources were determined to be statistically irrelevant.

**Table 1:** Key variables used in analysis.

Data Description	Data Link	Grain	Key Variables/Primary Key	Commentary
**CT DEEP** PM_2.5_	Sourced directly from CT DEEP	Hourly (1 Jan ’21–30 Jun ’22)	PM_2.5_, EST timestamp	97.5% of hours available
**PurpleAir – Gunntown**	PurpleAir - Gunntown	Hourly, at site (16 Apr ’21–30 Jun ‘22)	PM25_CF1_ug/m^3^, PM25_ATM_ug/m^3^, Temp_F, Humidity_%, Local timestamp	Operational periods, using CF = ATM, Missing initial observations
**PurpleAir – Long Meadow**	PurpleAir - Long Meadow	Hourly, at site (23 Mar ’21–30 Jun ‘22)	Same as Gunntown	Operational periods, using CF = ATM, Missing initial observations
**PurpleAir – Lake Zoar (“woti”)**	PurpleAir - Lake Zoar	Hourly, at site (1 Jan ’21–30 Jun ‘22)	Same as Gunntown	Operational periods, using CF = ATM
**Oxford Weather Data**	NOAA LCD	Hourly (1 Jan ’21–30 Jun ‘22)	Precipitation, Temp, RH, Pressure, Visibility, Wind Speed/Direction	Time stamp in local time, converted
**EPA CAMPD – CPV Towantic**	EPA Clean Air Mkts Prog Data	Hourly, at site (1 Jan ’21–30 Jun ’22)	Operating Level, Gross Load (MW), Heat Input (MM BTU), SO2 Mass (lbs), CO2 Mass (short tons), NOx Mass (lbs), by date	Variables highly correlated, Gross Load (MW) chosen
**Oxford Compressor Data**	Enbridge Infopost	Daily, at site (1 Jan ’21–29 Jun ’22)	First Cycle, Operationally Available Meter Capacity and Nominal throughput	MDTH/day (000's decathermals/day)
**Road Traffic Profile Data**	Gunntown-Chestnut Tree Rd Station ID = NAUG-164	3-day monitoring, (16–19 Mar ‘21) tested	Weekday profile of traffic count/% by class	Survey data over ∼3-day period, extrapolated to study timeframe
**Waterbury-Oxford Airport Take-off & Landing Data (TOLD)**	Flight Radar 24	Daily data, ∼Dec 2022 tested	Daily scheduled and tracked flights	1-month of data evaluated (Dec, ’22)

### 2.2 Analysis methods

#### 2.2.1 Analysis strategy

The Waterbury micro-area was chosen for this study owing to proximity to the CPV Towantic Energy Center, the Oxford “Algonquin” Compressor Station, the Waterbury-Oxford Airport, and available low-cost sensor data from PurpleAir's real-time map (https://map.purpleair.com). Hourly data were gathered from all potentially relevant sources over the period January 1, 2021–July 1, 2022. The 3 PurpleAir sensors included in this study (Gunntown, Long Meadow and Lake Zoar) produced output data at various times during this period, with the Lake Zoar sensor available for the greatest length of time (see charts in supplemental materials). The goal of the first part of this study is to estimate LCFs at each sensor using linear regression analyses to adjust PurpleAir PM_2.5_ output to be more aligned (calibrated) to that of the nearby CT DEEP Waterbury FEM Monitor, which required joining data by hourly timestamp. Given there was no low-cost sensor colocated with the reference grade monitor over the duration of this study, 3 PurpleAir locations nearby (8–16 km), and generally in the westerly direction from Waterbury, were chosen with the realization that there could be influence from intervening factors such as the ones to be explored in the study's second part (note that the Lake Zoar sensor is located to the southwest of the airport and energy enterprises). Once LCFs were determined, adjusted (calibrated) PurpleAir PM_2.5_ measurements were then joined by hourly timestamp to environmental (chiefly weather) and non-environmental (chiefly energy) data for logistic regression analyses. Joining hourly data from sources distributed over a micro-region implies a contemporaneous influence of factors on PM_2.5_ despite geo-spatial separation. Suggestions for incorporating time lags in more advanced time-series regression analyses are made toward the end of the paper.

Logistic Regression Modeling is a supervised learning approach used in statistical analysis when a dichotomous response variable is of interest, providing a linear relationship between log-odds and explanatory variables. As we are primarily concerned with elevated levels of calibrated PM_2.5_ as previously mentioned, the analysis incorporated an indicator outcome variable (0/1) for calibrated PM_2.5_ ≥ 12 μg m^−3^ measured from hourly data; that is, hourly calibrated PM_2.5_ was coded with a “1” if PM_2.5_ ≥ 12 μg m^−3^. The threshold of 12 μg m^−3^ is higher than the previously cited guidance by the World Health Organization (2021) and therefore values exceeding this threshold can be considered elevated. The threshold also provides a sufficient number of cases for feasibility of regression. Our hypothesis is that while the three PA sites are in the same micro-region, different estimated log-odds equations will be produced which may be suggestive of varying relative importance of the exogenous variables that prove to be the most explanatory or predictive of elevated PM_2.5_ periods (hours). Heteroskedastic-robust standard error estimation for parameters was deployed.

In addition, models were developed with and without an Auto-Regressive (AR) component (e.g., using the previous day's PM_2.5_ average), since an AR term may overwhelm the influence of other factors in terms of model fit and parameter estimation, and therefore developing models with and without this component is a prudent step. In the spirit of model validation, a reduced sample size (90%) was provided to the regressions to determine model/coefficient estimate stability (lower sample sizes were also evaluated).

Exogenous variables supplied to the logistic regression algorithm were identically defined at each site and are provided in the supplemental material. The selection of variables was based on literature review, availability of suitable data, and judgment/intuitive reasoning. Clearly other exogenous factors may play a role in determination of unacceptable PM_2.5_ periods, but associated data were unavailable or scant (e.g., air and roadway traffic data, mold spore counts, etc.). It should also be noted that many of these variables were measured contemporaneously with the PA PM_2.5_ outputs (aside from the L3H = “Last 3 Hours” and Yday = “Yesterday” variables). A model whose primary purpose is forecasting would naturally be endowed with a longer forecast horizon using time series analysis (e.g., an Auto-regressive Distributed Lag or “ARDL” model); this work is left for future research.

Univariate statistical analysis and data visualization informed variable transformations prior to building regression models for knowledge discovery. Namely, it was deemed that Temperature and Relative Humidity likely have non-linear relationships with levels of calibrated PM_2.5_ at each site. The following heuristically derived transformations were developed for use in logistic regression:

(1) $$Temp_{cF}=\left\{ \begin{array}{c} 1\;if\;Temp\_c\leq 32F\\ {\left(Temp\_c-60F\right)}^{2}\;otherwise \end{array}\right.$$

(2) $$RH\_cF={\left(RH\_c-60\%\right)}^{2}$$

in equations (1), (2), Temp_c and RH_c represent the Temperature and Relative Humidity, respectively, based on PA sensors primarily but imputed from Waterbury-Oxford airport data if missing (only at Gunntown for ∼ 5% of the observations).

#### 2.2.2 Data management

- Date formatting: given the CT DEEP Waterbury monitor records hourly data in Local Standard Time (Eastern Standard Time, or EST, in our case) format, it and the timestamps of other data sources used in this study required transformation to UTC for proper data joining (PurpleAir sensors record data in UTC format). Using Local Standard Time recorded by the CT DEEP Waterbury monitor is not recommended, as it does not follow Daylight Saving Time transitions, while other data sources do. This is evidently the case for all state monitors that follow Clean Air Act guidelines (per discussion with CT DEEP representatives).
- Data joins: for the purposes of analytics, data sources were joined using an outer join with the CT DEEP Waterbury monitor's calculated hourly UTC timestamp as the primary key. Missing data from other sources was handled in subsequent regression analyses (i.e., removed). A User Guide, all input data tables, supporting SQL code, resulting modeling input tables and associated Python code are provided in the supplemental materials. In addition, a Microsoft Access database, “**Waterbury Calibration Data v2.accdb**” which employs data management logic is provided for those who have a license.

## 3 Results

### 3.1 Exploratory data analysis (EDA) for calibration regressions

Prior to attempting no-intercept linear calibration regressions between the CT DEEP Waterbury monitor and each of the 3 PurpleAir sensors, data visualizations were employed. As noted earlier, the CT DEEP Waterbury monitor and PurpleAir sensors are based on different measurement technology, motivating the need for calibration of PurpleAir measurements to be in line with reference grade monitors. Moreover, anomalous PurpleAir measurements of PM_2.5_ above a value of 100 μg m^−3^ were considered outliers and removed from further analysis. PurpleAir sensors provide two output types: CF = 1 and CF = ATM, the former being an internal correction factor suggested for indoor use and the latter for outdoor use. While this study evaluated each, only results of the CF = ATM outputs are reported given this study is focused outdoors. It should also be noted that PM_2.5_ data do not generally follow a normal (Gaussian) distribution, and inferences based on such a distribution may not be valid.

Several observations emerge. First, there is a strong relationship – seemingly linear – between the CT DEEP Waterbury (WB) monitor's PM_2.5_ output and each PurpleAir (PA) sensor's (CF = ATM) PM_2.5_ output. Second, the intercept (expected value of WB PM_2.5_ when PA PM_2.5_ = 0) appears to be zero in each case – justifying a “no-intercept” calibration regression. Third, the slope of the relationship between WB measurements and each PA measurement set appears to be different – justifying separate (local) calibration regressions rather than a “one-size-fits-all” approach. Last, and perhaps most interestingly, a similar profile is evident in the plots of average PA PM_2.5_ measurements by Month of Year (MoY); a considerable spike is seen in the month of July and a lesser spike at the turn of the year. This phenomenon is also observed from WB data as well as PA sensors across the globe, with the exception of a few mostly arid locations (see Zhao et al. [30], for similar findings). A potential cause may be fungal spores [31]. Detailed study of this phenomenon is left for future research. Explanatory models derived from this study are assessed conventionally but also on the basis of their ability to reproduce the empirical monthly profile.

### 3.2 Local correction/calibration factors (LCFs; modeling WB reference grade monitor vs PA sensor outputs from 3 nearby, not colocated, locations)

There are 8 continuous FEM monitors in the state of Connecticut that measure PM_2.5_, one of which is in Waterbury [29]. CT DEEP has more recently deployed a colocated PurpleAir sensor at this site. Moreover, as previously mentioned, studies have aimed to produce a one-size-fits-all, broad-based, or nationwide correction factor (e.g., for woodsmoke as in Ref. [15]), which we have found to be unsupportable given the empirical analysis, and our secondary goal of finding local drivers of PM_2.5_ variation.

LCFs have been computed to adjust for the bias in raw data output from PurpleAir sensors, for reasons previously noted. The approach used in this study was to estimate a linear regression equation relating the CT DEEP Waterbury PM_2.5_ data paired with each PurpleAir sensor's PM_2.5_ data, assuming there is no intercept in the linear model. This is tantamount to assuming the CT DEEP monitor and PurpleAir sensors should record a measurement of zero PM_2.5_ when there is no ambient particulate matter measured by PA sensors, which is justified conceptually and empirically. Table 2 displays regression results and LCFs for each site.

**Table 2:** LCF Regression Results for PA sensors near Waterbury reference grade monitor; *data are not normally distributed.

Site	Gunntown	Long Meadow	Lake Zoar
**Observations**	8496	7394	11,920
**PA Coefficient**	0.7148	0.8309	0.6203
**Est. Equation**	WB_PM_2.5_ = 0.7148 × PA_PM_2.5_	WB_PM_2.5_ = 0.8309 × PA_PM_2.5_	WB_PM_2.5_ = 0.6203 × PA_PM_2.5_
95% Conf Int.*	(0.709, 0.721)	(0.820, 0.842)	(0.615, 0.625)
**90% Sample Est.**	0.7134	0.8302	0.6200
**R**^**2**^	0.863	0.761	0.838
**MAE**	3.00	3.80	3.13
**MSE**	17.11	23.09	17.72
**RMSE**	4.14	4.81	4.21

The following observations are made from this analysis. First, all regressions produce healthy adjusted R^2^, with 76%–86% of the variation in the CT DEEP Waterbury PM_2.5_ explained by regressing on site-specific PurpleAir PM_2.5_ measurements. Sensitivity checks produced similar estimated LCF values as shown in Table 2. These results are in alignment with research reviewed in the literature [32,19]. Second, site-specific LCF's vary significantly from site to site, with Lake Zoar's being the smallest at 0.6203, Gunntown's at 0.7148, and Long Meadow's the largest at 0.8309, supporting general findings made by other researchers [32,28], and which would be expected based on the empirical plots. Finally, although the data do not follow a normal distribution, the 95% confidence intervals (whose accuracy depends on the normality assumption) do not overlap, suggesting that the LCFs are *statistically* different – a conclusion that motivates the remainder of the paper. Finally, diagnostic statistics (MAE, RSME) are also consistent with earlier studies [32].

As this is not a controlled study, and not all potential contributing factors have been measured (e.g., fungal spores), care will be taken to avoid concluding causality when perhaps only suggestive correlations have been discovered and omitted variable bias may be a risk.

The *grain* of data used in estimation of LCFs matters, i.e., daily or hourly averages. As it is an objective to evaluate potential sources of variability using hourly data where practicable, we move forward with the LCF estimates based on hourly data. Based on the analyses in Table 2, we conclude that deployment of LCFs remains a prudent practice when sources of local variation are to be explored, as we do in the forthcoming evaluation of *Local Explanatory Regressions*. Regression analyses are based on PurpleAir output calibrated using LCFs from Table 2, e.g., for Gunntown analysis, PA PM_2.5_ values were multiplied by its unique LCF beforehand, etc.

### 3.3 Local explanatory regression (PA sensor calibrated PM_2.5_ vs explanatory variables)

Once each PurpleAir sensor has been calibrated to be consistent with (in terms of magnitude) the CT DEEP Waterbury reference grade monitor, we endeavored to understand potential drivers of elevated PM_2.5_ variation at each site, using candidate factors derived from Table 1. Our analysis paired data from each PA sensor with candidate explanatory factors by UTC hour. Time-based influence from key factors was evaluated by creating variables based on recent hours and also the average of the previous day. Regression analysis outside of a randomized controlled trial (RCT) will not confirm causality but rather indicate association (correlation in the case of linear models), and conclusions based on regressions not employing RCT are subject to confounding and omitted variable bias. To mitigate this risk, we explore customary validation methods.

Guidance from experts in the field (per discussion with CT DEEP) suggested attributing levels of or variation in PM_2.5_ by time of day and season of year, given the sun's influence during the day and through the year. In addition, as humans go about their typical work-week, a build-up of particulate matter arising from automobile exhaust, salt spray, dust, etc. may be influential. Accordingly, in addition to the factors in Table 1, the analysis included variables for Morning, Weekday, and Season/Month of Year. It was anticipated that inclusion of Temperature and Relative Humidity as correlates may account for the influence of quotidian or seasonal variation arising from these time factors and would render them superfluous.

Univariate analysis, exploring each variable's relationship with each PurpleAir sensor's calibrated PM_2.5_, was performed, using decile means plots associated with each sensor site. Decile means are particularly useful when scatterplots fail to reveal useful patterns in the data; deciles are calculated for the explanatory variable and the mean values of PM_2.5_ (calibrated) are plotted against the mean values of the explanatory variable within each decile. These values are connected for visual interpretation and a linear trend is overlayed but not implied. The purpose of this analysis, found in the supplemental material, is to inform subsequent regression modeling in terms of variable transformation and model formulation.

**Fig. 2:**
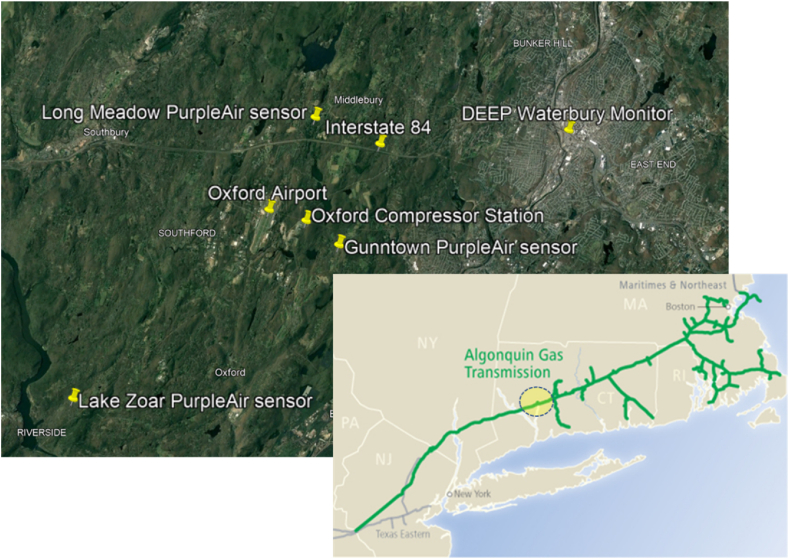
Region of PM_2.5_ Study, with Measurement Locations and Noteworthy Features (inset – “Algonquin” Fossil Gas Pipeline; source: Enbridge

Several observations are made:

1. **Temperature** (degrees Fahrenheit) displays a somewhat similar *non-linear* relationship with calibrated PM_2.5_ at each site. The linear trendline at Gunntown displays a positive slope. The pattern suggests a different relationship at the freezing point (32F) than for higher values. Moreover, a parabolic relationship with a vertex at ∼60F is exhibited at values above freezing.
2. **Relative Humidity** (% saturation) also has displays a non-linear relationship at each site, with a general positive trend as values increase, although there is a concave, perhaps parabolic, relationship with a vertex near 60%.
3. **Wind Speed** (mph, measured at the Waterbury-Oxford Airport) displays a dramatic negative relationship at each site; conceptually this is reasonable given that wind would tend to scatter/mix particulate matter.
4. Gross Load at the **CPV Towantic Energy Center** (MW, measured at the center) displays a similar relationship at each site that is generally positive but potentially non-linear. There is a high concentration of values in the 400 MW neighborhood.
5. Throughput of the **Oxford “Algonquin” Compressor Station** (MDTH/day, measured at the station) displays slightly different trends by site that are also potentially non-linear. Interestingly, there appears to be a local minimum of average PM_2.5_ at a throughput of 1200 MDTH/day at each site. The Gunntown PA site is approximately 1.4 km away from the compressor station in the easterly direction (see Fig. 2).

**Precipitation** (inches, measured hourly at the Waterbury-Oxford Airport) generally has a negative relationship at each site, also conceptually sensible as precipitation tends to mitigate PM [33]. Data were not amenable to decile means analysis given the relatively low amounts of rainfall and snowfall during the study period at any given time. Instead, boxplots were used for graphical display and evaluation.

**Roadway** and **Airway Traffic** data were also explored; it was hypothesized (as is suggested in the literature by Kim et al., [21]) that there is a positive relationship between vehicle traffic and PM_2.5_ levels. However, owing to the limited nature of the available data (3-day and 1-month periods for road and air traffic, respectively), relative traffic influences were left for future research.

In summary of our univariate analyses, there appear to be factors with useful value in terms of explaining variation in calibrated PurpleAir PM_2.5_ observations at each site. Some factors exhibit non-linear relationships based on data visualizations. Given our primary interest is in understanding what drives higher values of PM_2.5_ and if different relationships exist between PM_2.5_ measured at each site, a reasonable next step is to employ regression techniques by site that allow for non-linear relationship exploration and reliably explain higher instances of calibrated PM_2.5_ observations. To that end, we next model these higher values (established at ≥ 12 μg m^−3^ at each site) using Logistic Regression Modeling.

### 3.4 Logistic regression results

Table 3 displays the results of 3 regression trials for each site: a) no auto-regressive (“no AR”) component, b) AR (with AR component), and c) 90% sample (with AR component).

**Table 3:** Logistic regression results.

Explanatory Variable	Gunntown			Long Meadow			Lake Zoar		
	no AR	AR	90% Sample	no AR	AR	90% Sample	no AR	AR	90% Sample
Intercept	55.9111	47.7051	46.0071	54.7171	49.3009	46.5489	11.9694	5.4351*	5.0246*
CPV_nom	0.0013	0.0011	0.0010	0.0007	0.0007	0.0005*	0.0010	0.0010	0.0011
OA_nom	0.0018	0.0014	0.0013	0.0037	0.0032	0.0033	0.0034	0.0024	0.0025
CPV_change	0.0045	0.0039	0.0041	0.0046	0.0040	0.0038	0.0029	0.0019	0.0020
Temp_cF	0.0026	0.0024	0.0024	0.0016	0.0015	0.0016	0.0018	0.0017	0.0017
RH_cF	−0.0020	−0.0021	−0.0022	−0.0017	−0.0016	−0.0017	−0.0023	−0.0024	−0.0023
HrlyViz	−0.0940	−0.0933	−0.0969	−0.1018	−0.0860	−0.0867	−0.0710	−0.0710	−0.0689
HrlyWindSpd	−0.0675	−0.0702	−0.0744	−0.0403	−0.0491	−0.0522	−0.0908	−0.1001	−0.1013
HrlyPress	−1.8858	−1.5876	−1.5294	−1.9628	−1.7553	−1.6601	−0.4453	−0.2110*	−0.2034*
WindNW	−0.8918	−1.1247	−1.1893	−1.0877	−1.1964	−1.1228	−0.9250	−1.0315	−1.0746
WindSW	−0.2465	−0.2607	−0.3452	N/A	N/A	N/A	N/A	N/A	N/A
WindNE	−0.6745	−0.7466	−0.8022	−0.7295	−0.8063	−0.8033	−0.5613	−0.5861	−0.5983
L3H_precip	−7.3076	−6.5557	−6.7752	−8.7541	−7.6337	−7.3393	−11.9997	−11.9143	−11.3318
L3H_Temp	N/A	N/A	N/A	0.0274	0.0287	0.0314	N/A	N/A	N/A
L3H_RH	N/A	N/A	N/A	0.0138	0.0177	0.0180	N/A	N/A	N/A
L3H_WindSpd	−0.1099	−0.1377	−0.1347	−0.1165	−0.1360	−0.1387	−0.1280	−0.1511	−0.1524
Yday_RH	−0.0270	−0.0451	−0.0449	−0.0494	−0.0723	−0.0769	−0.0292	−0.0429	−0.0418
Yday_WindSpd	−0.2640	−0.1712	−0.1569	−0.3369	−0.2699	−0.2726	−0.2308	−0.0823	−0.0805
Yday_Precip	−1.0979	−0.8936	−0.8632	0.4887	0.7250	0.7242	−0.9358	−0.7307	−0.7046
Yday_PM2.5	N/A	0.0799	0.0828	N/A	0.0782	0.0796	N/A	0.0821	0.0822
KS, ROC	0.5429,0.8512	0.5961,0.8786	0.6000,0.8792	0.5980,0.8766	0.6544,0.8886	0.6514,0.8909	0.5874,0.8752	0.6462,0.8949	0.6402,0.8943

Models are evaluated using various standard techniques including Kolmogorov-Smirnov (KS), Receiver Operating Characteristic (ROC) and Variance Inflation Factors (VIFs). The modeling process was essentially backward elimination, with each site's “no AR” model presented with the full complement of exogenous factors, and variables subsequently eliminated if statistically insignificant (p-value = 0.05), or their VIF was greater than 5. Once the “no AR” model was derived, the AR component was added and coefficient magnitudes and signs reviewed. Finally, the model was redeveloped with the AR component with a reduced sample size, and again coefficient magnitudes and signs reviewed.

Red font in Table 3 signifies a *positive* association with log-odds of elevated PM_2.5_, while green signifies a *negative* association. An asterisk (*) indicates that the variable was not significant at the 0.05 level.

Some take-aways from Table 3:

1. Goodness of fit metrics are good – all models produce acceptable levels of KS and ROC.
2. Parameter estimates by site are largely stable after a) inclusion of AR term (Yday_PM_2.5_), and b) 10% sample reduction (also evaluated at 20% and 30%). The model henceforth on which to focus is the AR model at each site.
3. Parameter estimate magnitude is a function of underlying variable units.
4. Parameters are interpreted, with some exceptions, as the resulting change in log-odds from a positive 1-unit change in the variable, *ceteris paribus*; for example, increasing L3H_precip (precipitation levels over the last 3 h) by 1 unit (i.e., 2.54 cm) at Gunntown would change the log-odds by −7.31 and therefore change the odds of elevated PM_2.5_ by exp (-7.31)-1 = −0.99 or 99% reduction, *ceteris paribus.*
5. The interpretation of Temp_cF and RH_cF coefficients is more complex, as these are functions of Temperature and Relative Humidity. The positive coefficient on Temp_cF implies that for levels above freezing, Temperature has a convex quadratic relationship with PM_2.5_ with a vertex at 60F. A negative coefficient on RH_cF implies that Relative Humidity has a concave quadratic relationship with PM_2.5_ with a vertex at 60%. This is consistent with the data visualizations.
6. Generally, the energy-related factors have a positive association with log-odds and meteorological factors have a negative association. A counterexample to this is the association with the previous day's average precipitation (Yday_Precip) at Long Meadow, perhaps owing to the increased elevation above sea level at this site.

**Fig. 3:**
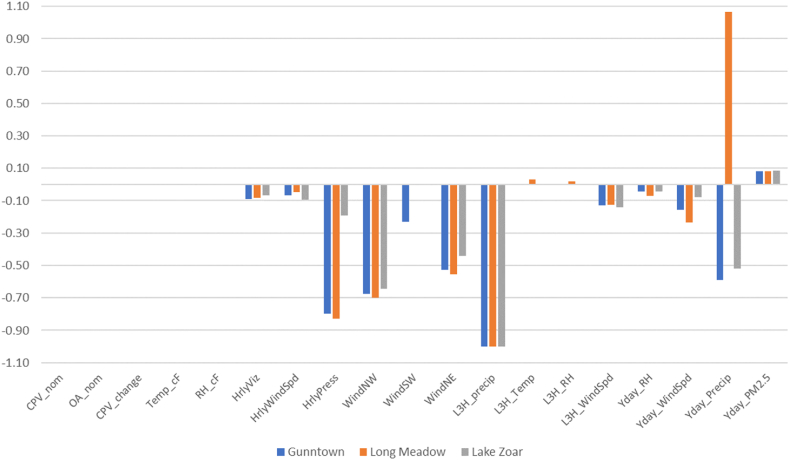
Relative Influence (in odds of PM2.5 ≥ 12 μg m^−3^) of a positive 1 unit factor change.

**Fig. 4:**
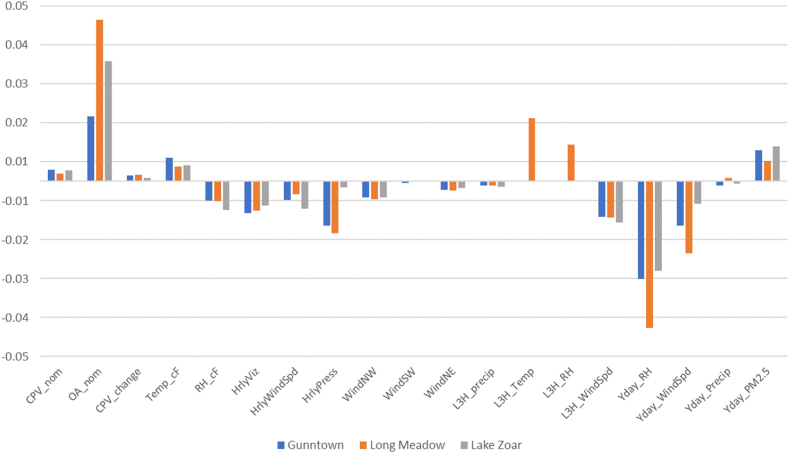
Relative Influence (in odds of PM2.5 ≥ 12 μg m^−3^) of a positive 1% factor change.

Fig. 3 provides a graphical display of the influence of a 1-unit change in each variable on the log-odds of an elevated PM_2.5_ period. It suggests that the energy variables have negligible influence on the odds of elevated PM_2.5_, while wind and precipitation are substantial. Clearly a 1-unit change, *ceteris paribus*, can be large for some variables (e.g., precipitation in inches) but small for others (e.g., Gross Load in MW). Fig. 4 provides a graphical display of the influence of a *1% change* in each variable (from the mean) on the odds of an elevated PM_2.5_ period; 1% is well below the Coefficient of Variation (CV) for each variable in the models, e.g., the CV for OA_nom in the Gunntown log-odds model is 20%, or 20× the change being evaluated in Fig. 4, and is the lowest CV produced.

Fig. 4 seems a more realistic evaluation of relative factor influence for the quantitative factors. Take-aways from this analysis are:

1) A 1% change (from average level) in previous day's RH and Wind Speed have the largest *negative* association with odds of an elevated PM_2.5_ period, *reducing* odds by 1–3% on average.
2) A 1% change in previous day's RH has the greatest negative impact on odds at ∼3–4%, with greatest influence at Long Meadow.
3) A 1% change in compressor throughput (MDTH/day) *increases* odds by 2–5% on average, with greatest influence at Long Meadow.
4) A 1% change in energy production (MW) only *slightly increases* odds.

Greater changes in the input variable may produce greater changes in log-odds, albeit *mutatis mutandis*. Based on Fig. 4 and analysis of likelihood predictions above 90%, in situations for which compressor throughput is elevated (>1300–1400 MDTH/day), at Temperatures generally lower than 60F but above freezing, at Relative Humidity near 60% (but higher on average than the previous day), low/no Wind Speed, low/no Precipitation, and when the previous day's average PM_2.5_ was elevated, then persistently higher levels of PM_2.5_ are assured and harmful conditions are present.

### 3.5 Model output analysis

The supplemental material contains graphical displays of model output (“prediction”) by site compared with actual data. Plotted are the average actual level proportion of hours of elevated PM_2.5_ by month of year, compared to the average of hourly predicted probabilities. An investigation of mold/fungus spore contribution to PM_2.5_ may prove useful in closing the underprediction manifested in July of each year.

## 4 Discussion and conclusions

In the present case study, relevant to the micro-area around Waterbury, Connecticut, we found the following:

Energy-related variables, temperature, and previous day's PM_2.5_ have a *positive* association with log-odds (supporting *a priori* hypotheses). That is, the CPV/OA energy complex is associated with particulate matter in proportion to its use, with compressor station throughput having a markedly larger positive impact than energy production and many meteorological variables under a modest positive change in input levels. It does not appear to be true that the influence of the CPV/OA energy complex would be more manifest at Gunntown than the other two PA sites (Gunntown has the least positive impact based on compressor throughput). This indicates one or more of the following: combusted fossil gas from the compressor distributes and settles across the region, or end-user combusted fossil gas is present at/nearby each sensor, or throughput of fossil gas is a covariate/confounded with unmeasured phenomena such as use of wood or fuel oil heating. Nevertheless, monitoring air quality at the energy center is recommended.

Other meteorological variables (RH, Visibility, Wind Speed, Relative Wind Direction, and Precipitation) generally have a *negative* association with log-odds, other than at Long Meadow – perhaps owing to its elevation (generally supporting *a priori* hypotheses). Temperature and Relative Humidity have a non-linear association with odds of elevated PM_2.5_, as described earlier in the paper; the previous day's Relative Humidity at the Long Meadow sensor has the greatest *negative* influence on odds, while the other two sites exhibit similar behavior. Wind Speed has a *negative* impact on odds, as expected, while northerly winds have a *negative* association *relative* to southerly winds. Use of these meteorological factors is critical to properly attributing potential drivers of elevated PM_2.5_.

Finally, as anticipated, precipitation has the largest influence under a positive 1-unit change (generally *negative*), while Oxford “Algonquin” throughput (*positive*), Relative Humidity and Wind Speed (*negative*) have the largest influence under a positive 1% change (over average factor levels).

Generally, we find that low-cost PurpleAir sensors are quite useful in supporting analysis of ambient conditions on PM_2.5_. Calibrations to non-colocated reference grade monitors are more precise if done on a micro-level and not macro-level (e.g., by region, state, nation, etc.). For example, Gunntown's LCF is 0.7148, Long Meadow's LCF is 0.8309, and Lake Zoar's LCF is 0.6203, despite being within 6–16 km of each other. When exogenous data are available, even if non-colocated to a sensor, they can be useful to understand PM_2.5_ variation; a decile means analysis approach is particularly illuminating.

When the objective is to understand potential drivers of higher levels of PM_2.5_, a logistic regression approach has utility. Modeling identifies associations between key exogenous variables and log-odds of elevated PM_2.5_. Incorporating previous day's average PM_2.5_ improves modeling (and has a *positive* influence on log-odds), indicating autoregressive modeling may be justified.

Based on the relative straightforward application of low-cost sensor data in this paper, admittedly based on a “convenience” selection of PurpleAir sensors and their locations, a more controlled study by state regulators with sensors placed at strategic locations throughout the state would be highly beneficial. The authors provide all content of the present study in the supplemental materials to anyone who wishes to replicate, apply or extend the work.

More monitoring by CT DEEP, using low-cost PM_2.5_ sensors such as PurpleAir, placed at strategic locations throughout the state and also organizing private sensor deployment, data aggregation and analysis, would supplement the current state Air Quality plan considerably. Moreover, the monitoring (and alerting) on raw/hourly data, with a fixed threshold (e.g., calibrated hourly PM_2.5_ ≥ 12 μg m^−3^) throughout the state/region, that does not get altered from year to year as EPA mandates change, is highly recommended. As discussed earlier, even short-term exposure to PM_2.5_ is linked with very real and negative human health impacts, which should motivate local monitoring and reporting of attributable factors on a more frequent basis, e.g., hourly. Accelerating energy transition to electrification could eliminate a substantial portion of elevated PM_2.5_.

The connection between air pollution, health, and climate change can be used to benefit humanity: reductions in burning fossil fuels reduces air pollution and slows global heating, adding years to global life expectancy [34,35]. The science is clear and the solution to the epidemic of preventable air pollution deaths must include public health policies that reduce or eliminate fossil fuel emissions and facilitate the transition to clean energy [2,6].

The limitations of this study are as follows:

a) PurpleAir sensors used in this study were selected based on proximity to a reference grade monitor (Waterbury) and an operating energy center (Oxford). While the approach should be transferrable to any location, the specific conclusions may not be.
b) Hypotheses regarding calibration factors hinge on various assumptions (e.g., Normality, Independence) which are likely not met. Caution should be used when interpreting confidence interval estimates.
c) The “attribution” analysis was done chiefly on data contemporaneous with PM_2.5_ measurements at each sensor, although some of the hypothesized influencers of PM_2.5_ are not measured in a colocated fashion. An approach using time lags, such as Autoregressive Distributed Lag modeling (ARDL) should prove useful. Our objective was a preliminary attempt at attribution of higher levels of PM_2.5_ to influencers measured contemporaneously (same hour), recently (previous 3 h) and historically (previous day) and the analysis suggests that ARDL could have benefits. Clearly a spatial-temporal modeling approach is applicable, as suggested by other authors.
d) Similarly, it would be most beneficial to gather weather-related data at each sensor rather than use data from a nearby source (e.g., Waterbury-Oxford Airport) as a proxy for weather data at each site.
e) Modeling of Temperature and RH based on decile means was heuristic (e.g., the 60F and 60% vertices and a parabolic assumption) and could be modeled more analytically.
f) Air and Roadway Traffic are likely positive influencers but lack of data availability prohibited their use. Roadway traffic data are typically available over a very short time horizon (a few days), but these data didn't justify continued use based on preliminary conclusions – a *negative* association with PM_2.5_. Take-off and Landing (TOLD) data are available on a daily basis at a substantial cost. Based on a very limited time horizon (a month) for publicly available data, analysis did not prove conclusive. A more detailed analysis is suggested.

## Statements and acknowledgements

The authors wish to thank Nathan Phillips, Ph.D. and Robert Ackley for their valuable contributions to this paper.

## Author contribution statement

Tim Keyes, Ph.D.; Martha Klein: Conceived and designed the experiments; Performed the experiments; Analyzed and interpreted the data; Contributed reagents, materials, analysis tools or data; Wrote the paper.

Rea Domingo, M.S.; Samantha Dynowski; Royal Graves; Melissa Leonard; John Pilgrim; Alison Sanchirico; Kate Trinkaus: Analyzed and interpreted the data; Contributed reagents, materials, analysis tools or data; Wrote the paper

## Data availability statement

Data associated with this study has been deposited at https://github.com/TKKeyes/HELIYON-D-23-18430.git.

## Declaration of competing interest

The authors declare the following financial interests/personal relationships which may be considered as potential competing interests:As per journal requirements, it should be noted that lead author Tim Keyes was a paid consultant, commissioned by the Sierra Club Connecticut for this work.
