# Supplementary material for: Low-cost PM2.5 sensors can help identify driving factors of poor air quality and benefit communities
Source: Heliyon. 2023 Sep 6;9(9):e19876. doi: 10.1016/j.heliyon.2023.e19876 (PMC10559280; doi:10.1016/j.heliyon.2023.e19876)
Supplement: Multimedia component 2 [file mmc2.docx]

**Supplemental Content for**

**Low-Cost PM_2.5_ Sensors Can Help Identify Driving Factors of Poor Air Quality and Benefit Communities**

Tim Keyes^a,b,^[[1]](#footnote-1)^,^[[2]](#footnote-2)^^ , Rea Domingo^b^, Samantha Dynowski^c^, Royal Graves^c^, Martha Klein^c^, Melissa Leonard^c^, John Pilgrim^c^, Alison Sanchirico^c^, Kate Trinkaus^c^

^a^ Evergreen Business Analytics, LLC, U.S.A.

^b^ Sacred Heart University, U.S.A.

^c^ Sierra Club Connecticut, U.S.A.

| Lat = 41.55046d N, Long = 73.04365d W, 269 feet above sea level |
| --- |
| Established 1975 |
| Measures PM_2.5_ (FEM), PM_10_, Wind Speed, Wind Direction, Temperature (FEM = [Federal Equivalent Method](https://www.epa.gov/air-research/epa-scientists-develop-and-evaluate-federal-reference-equivalent-methods-measuring-key)). |
| The site uses an FEM Teledyne API T640 monitor for PM_2.5_ mass concentration. The Teledyne API Model T640 with T640X Option is a real-time, continuous PM mass monitor that uses scattered light spectrometry (using 90 degree white-light scattering with polychromatic LED) for measurement of PM_2.5_, and the T640X Option measures 2.5, 10, and coarse PM. The sample rate is 5 LPM (T640) or 16.67 LPM (T640X). |
| 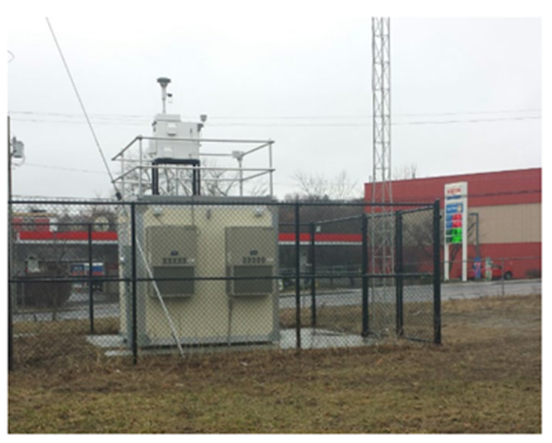  Waterbury Bank Street Monitor |

*CT DEEP Waterbury Bank Street Monitor (Source: CT DEEP, 2022)*

| Gunntown: Lat = 41.48283d N, Long = 73.1034d W, 516 feet above sea level |
| --- |
| Long Meadow: Lat = 41.513873d N, Long = 73.138823d W, 657 feet above sea level |
| Lake Zoar: Lat = 41.399733d N, Long = 73.167379d W, 321 feet above sea level |
| Measures PM_1.0_, PM_2.5_, & PM_10_ for indoor & outdoor use, Temperature, Relative Humidity. |
| PurpleAir PA-II-SD devices use 2 PMSX003 sensors, specifically the Plantower PMS5003. These operate using a class 3a/3R laser and a detector plate to measure PM. |
| A fan draws in air through the path of a laser beam, and passing PM reflects light onto the detector which measures the reflected light pulse. The duration of the pulse indicates the size of the PM, and the number of pulses is used to determine the PM concentration. The 2 PMS5003 sensors measure PM in real time, with each one alternating 5-second readings averaged over 120 seconds. |
| Wifi-enabled. See [PurpleAir map](https://map.purpleair.com/1/mPM25/a0/p604800/cC0#10.63/41.4579/-73.1263). |
| 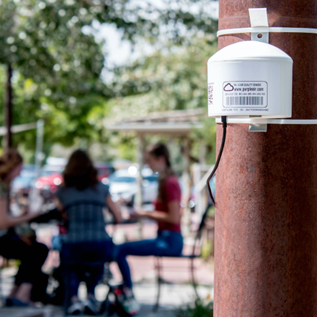  A typical PurpleAir-II-SD sensor |

*PurpleAir Sensors (Source: https://www2.purpleair.com/products/purpleair-pa-ii)*

The following tables provide selected data visualizations of paired hourly data (i.e., when both the CT DEEP and PurpleAir outputs were available) – outliers have not been removed.

| **Sensor Location** | **WB (y-axis) vs PA Site (x-axis) – Hourly** PM_2.5_ |
| --- | --- |
| Gunntown | 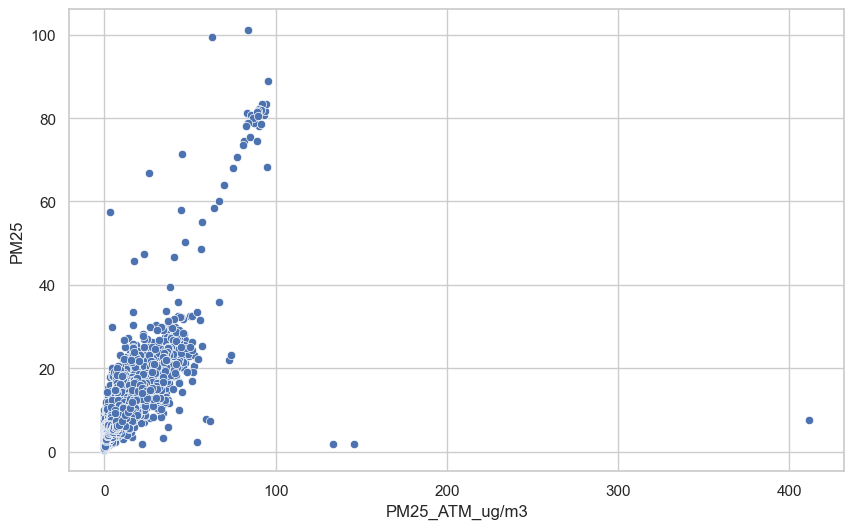 |
| Long Meadow | 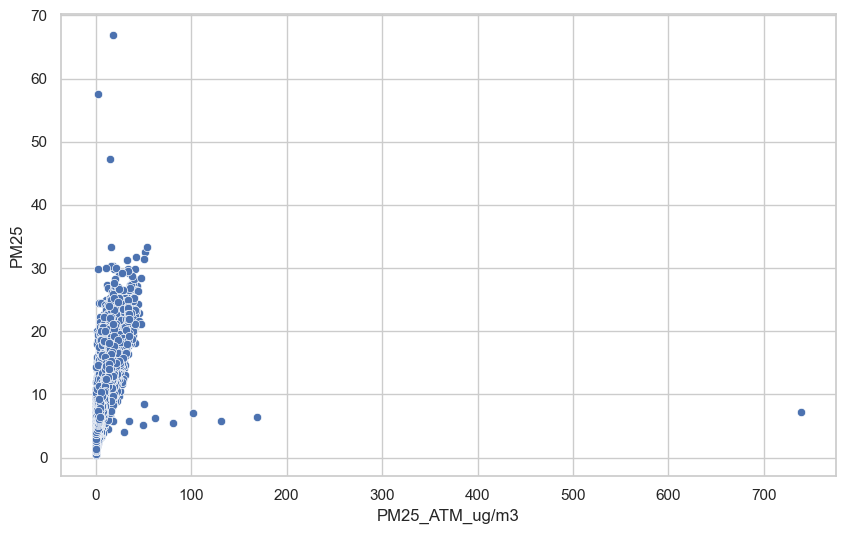 |
| Lake Zoar | 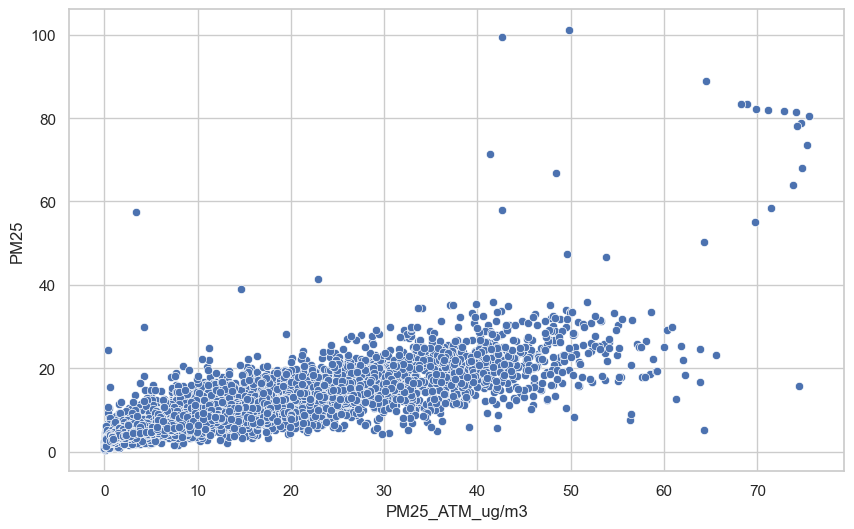 |

*Data visualizations for PurpleAir sensors*

| **Sensor Location** | **WB FEM Monitor and PA Site Time Series – Avg** PM_2.5_ |
| --- | --- |
| Gunntown | 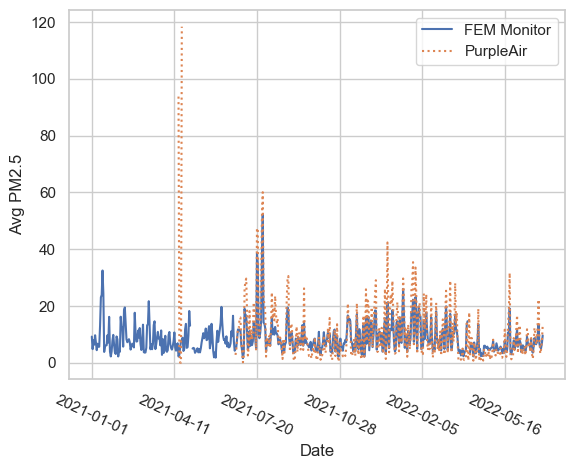 |
| Long Meadow | 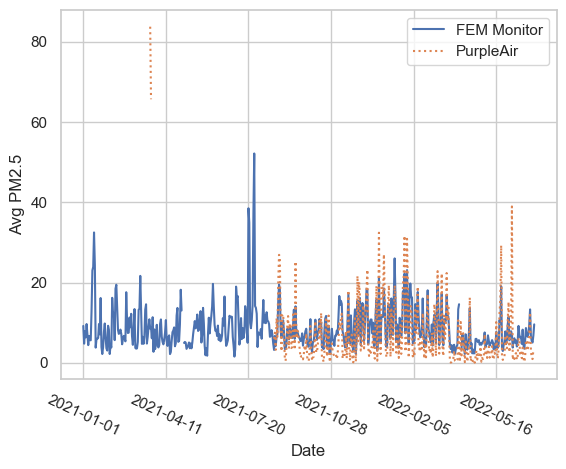 |
| Lake Zoar | 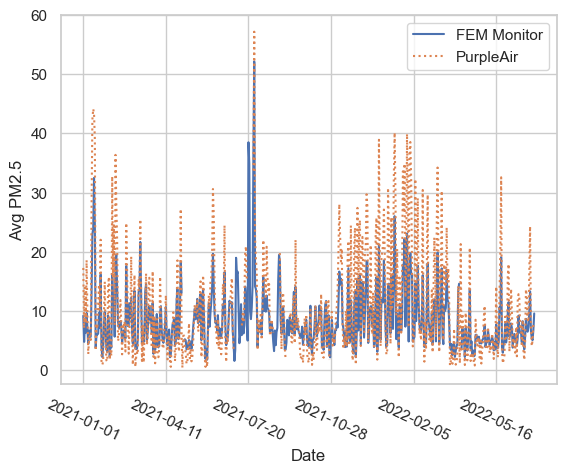 |

| **Factor** | **Gunntown** | **Long Meadow** | **Lake Zoar** |
| --- | --- | --- | --- |
| Temp (F) | 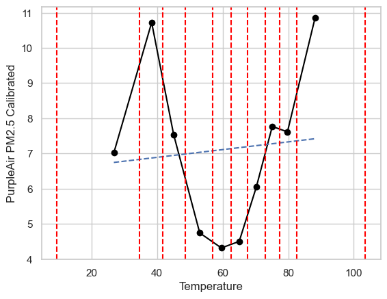 | 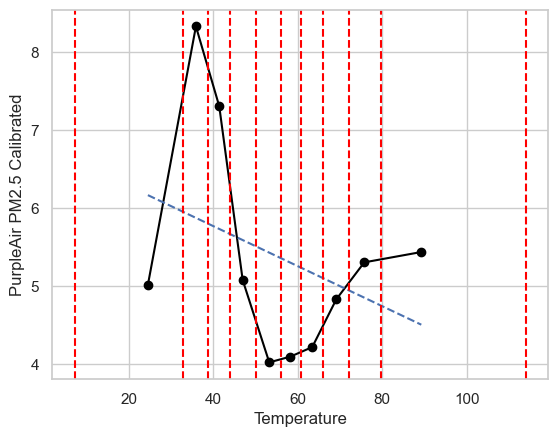 | 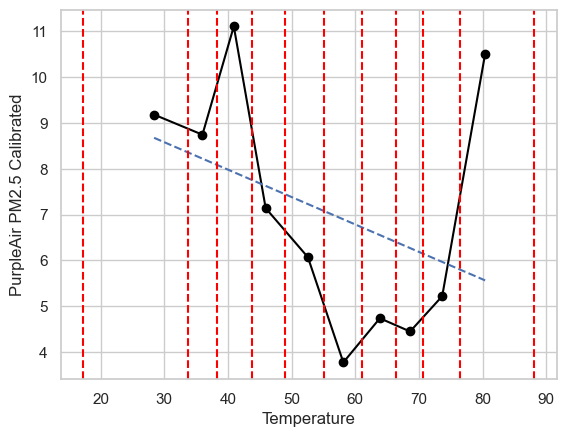 |
| RH (%) | 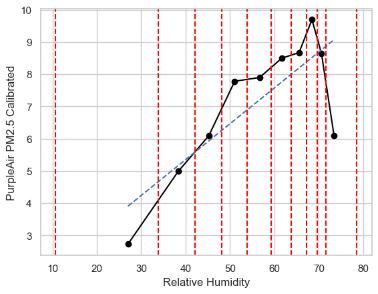 | 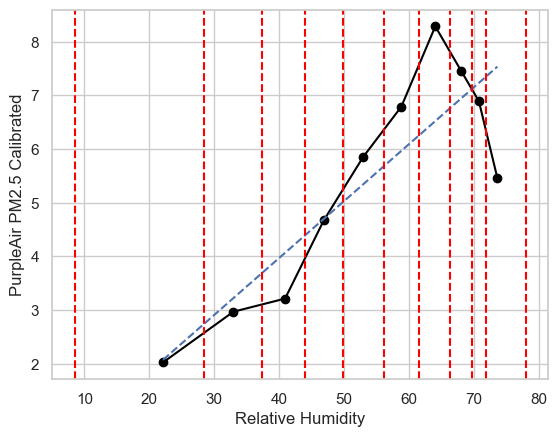 | 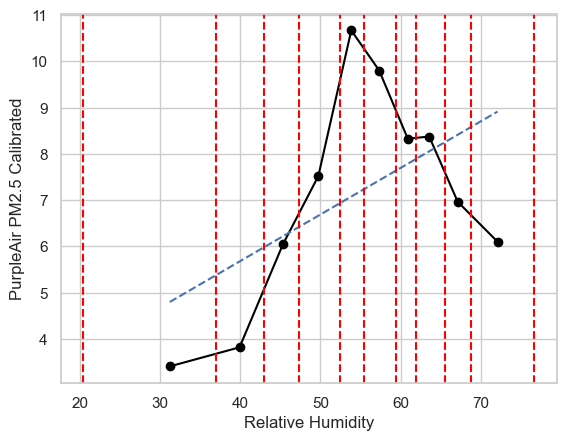 |
| Wind Speed (mph) | 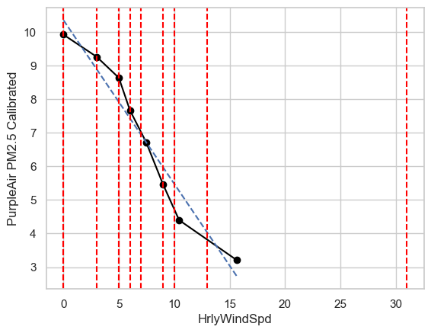 | 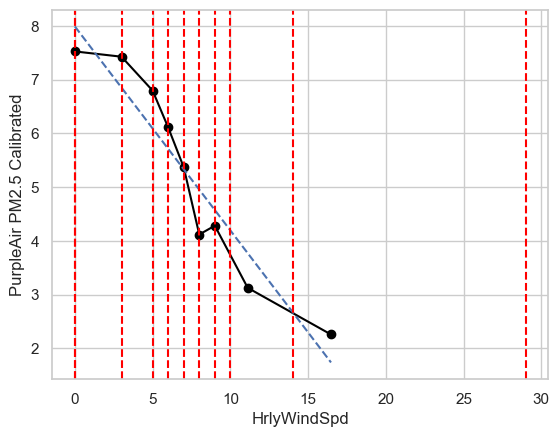 | 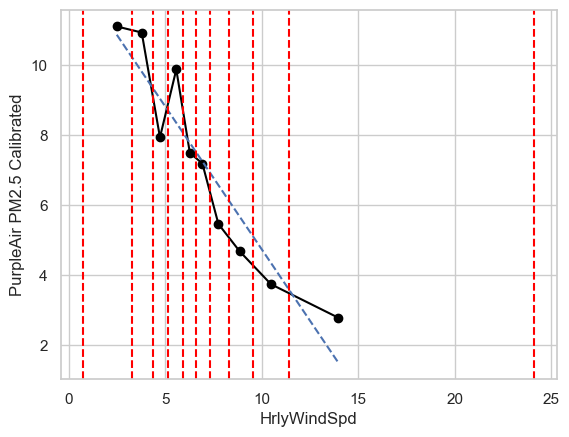 |
| CPV Towantic Gross Load (MWH) | 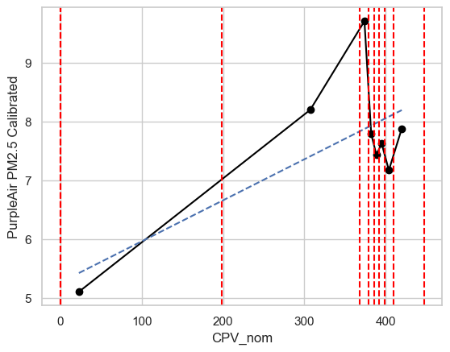 | 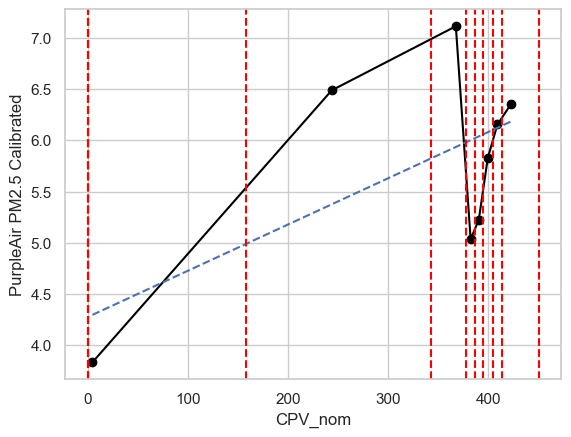 | 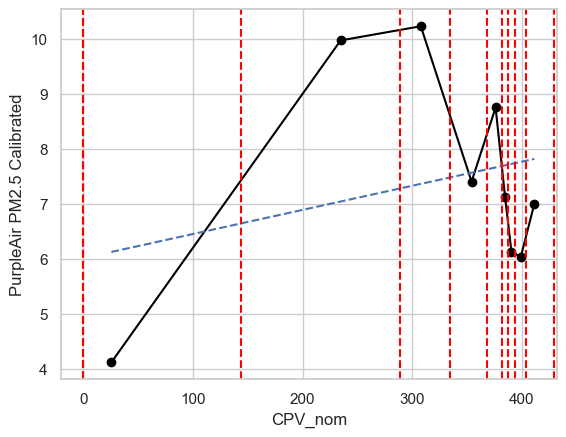 |
| Compressor through-put (MDTH/day) | 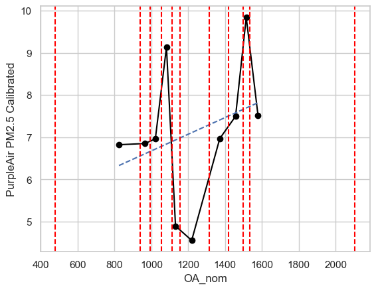 | 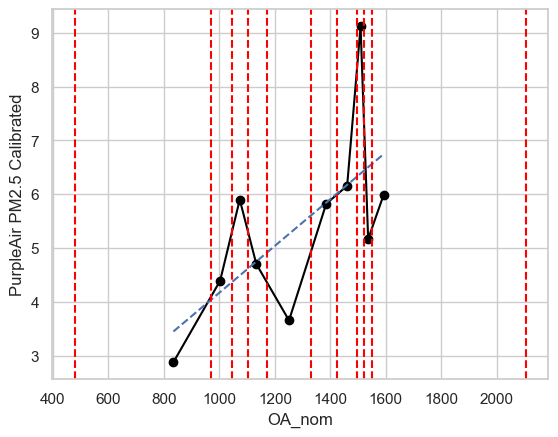 | 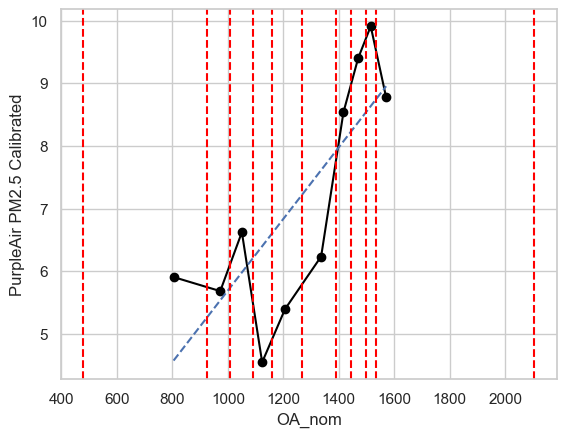 |

*Univariate decile means of key variables vs calibrated PM_2.5_ by site*

| **Sensor Location** | **Calibrated Purple Air PM_2.5_ vs. Precipitation Indicator** |
| --- | --- |
| Gunntown | 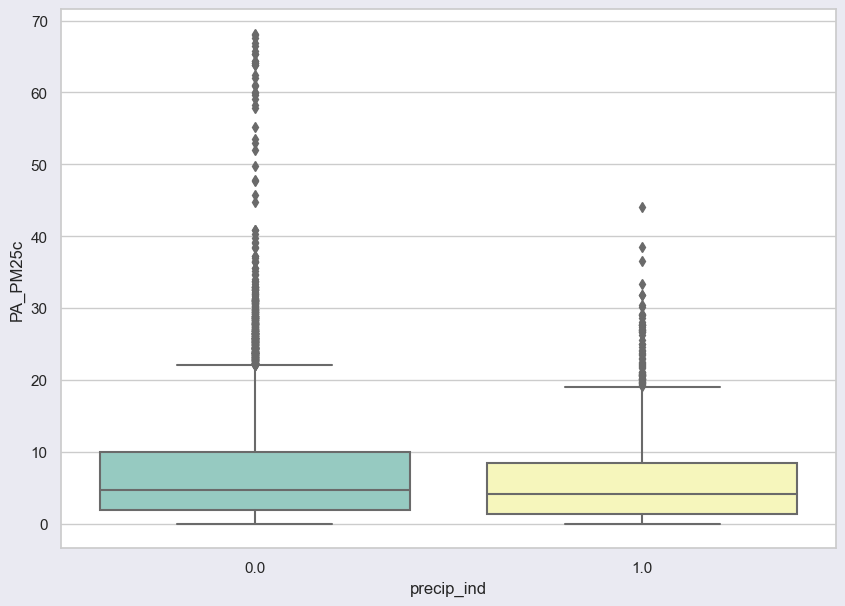 |
| Long Meadow | 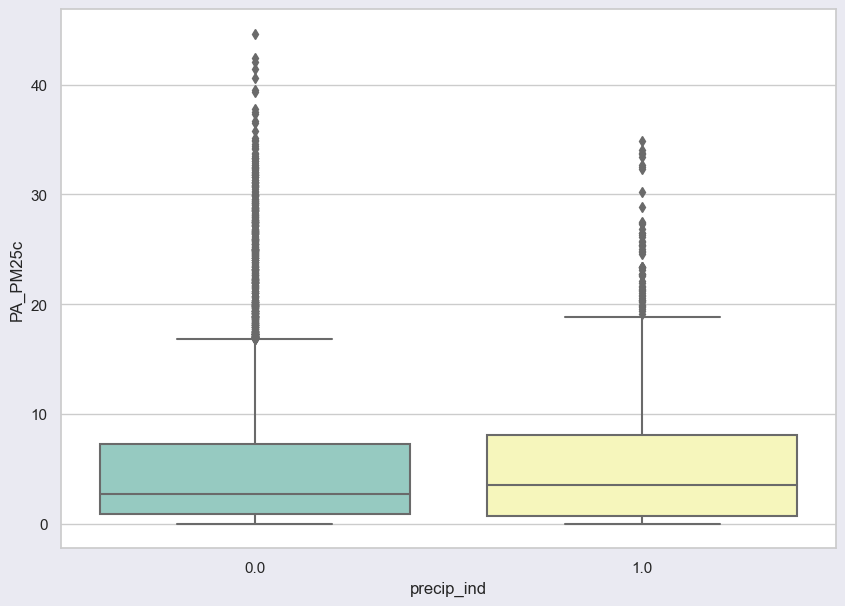 |
| Lake Zoar | 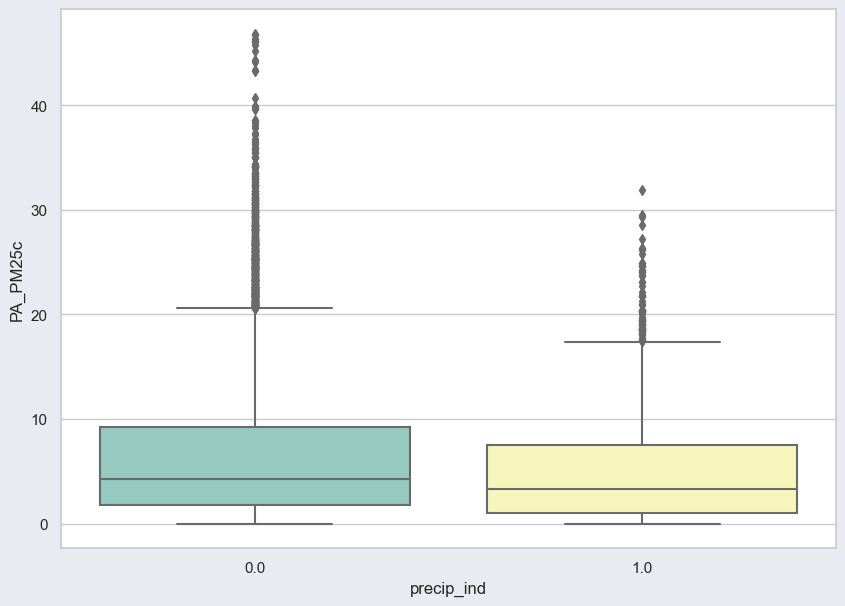 |

*Influence of Last 3 Hrs Precipitation on Calibrated PM_2.5_ (precip_ind=1 implies precipitation in last 3 hours)*

| **Exogenous Variable** | **Granularity** | **Definition** |
| --- | --- | --- |
| **CPV_nom** | Hourly data | Nominal value for CPV Towantic Energy Center Gross Load (MW) measured hourly |
| **OA_nom** | Daily data | Nominal value for Oxford “Algonquin” Compressor station throughput (MDTH/day) measured daily |
| **CPV_change** | Hourly data | Magnitude of change in CPV Towantic Gross Load (MW) over last 3 hours |
| **OA_change** | Daily data | Magnitude of change in Oxford “Algonquin” throughput (MDTH/day) measured over last 3 hours |
| **Temp_c** | Hourly data | Temperature (degrees F) primarily at each PA site, with missing values imputed from the Waterbury-Oxford Airport (WOA) data (Gunntown only; 5.4% missing), measured hourly; a non-linear function of Temperature was used given the findings from decile means |
| **RH_c** | Hourly data | Relative Humidity (%) primarily at each PA site, with missing values imputed from WOA data; a non-linear function of RH was used given the findings from decile means |
| **Morning** | Hourly data from UTC timestamp | Indicator (0/1) variable for local morning |
| **Weekday** | Daily data from UTC timestamp | Indicator variable for weekday vs weekend |
| **HrlyPrecip** | Hourly data | Total precipitation at WOA measured hourly (inches) |
| **HrlyViz** | Hourly data | Visibility at WOA measured hourly (miles) |
| **HrlyWindSpd** | Hourly data | Wind speed at WOA measured hourly (mph) |
| **WindNW** | Hourly data | Indicator for WOA wind direction generally from the northwest |
| **WindSW** | Hourly data | Indicator for WOA wind direction generally from the southwest |
| **WindNE** | Hourly data | Indicator for WOA wind direction generally from the northeast |
| **L3H_precip** | Hourly data | Leading 3 hours total precipitation at WOA measured hourly (inches) |
| **L3H_Temp** | Hourly data | Leading 3 hours of temperature (degrees F) |
| **L3H_RH** | Hourly data | Leading 3 hours of relative humidity (%) |
| **L3H_WindSpd** | Hourly data | Leading 3 hours of WindSpd (mph) |
| **Yday_Temp** | Daily data | Average temperature one day prior |
| **Yday_RH** | Daily data | Average relative humidity one day prior |
| **Yday_WindSpd** | Daily data | Average wind speed one day prior |
| **Yday_Precip** | Daily data | Total precipitation one day prior |

*Exogenous variables used in Regression*

| **Sensor Location** | **Predicted vs Actual PM_2.5_ by Month of Year** |
| --- | --- |
| Gunntown | 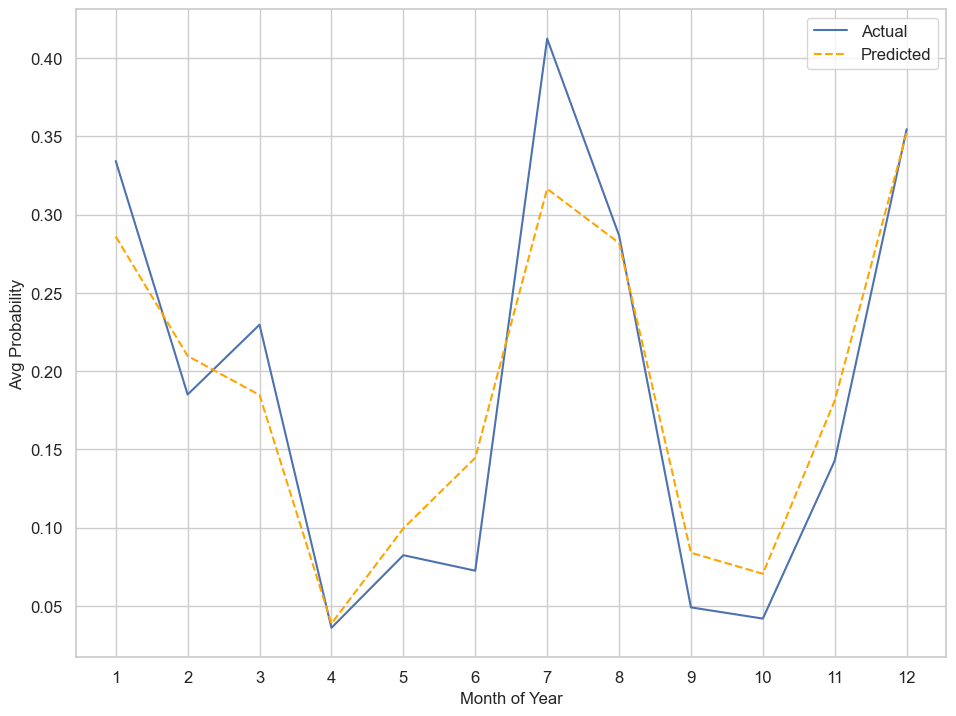 |
| Long Meadow | 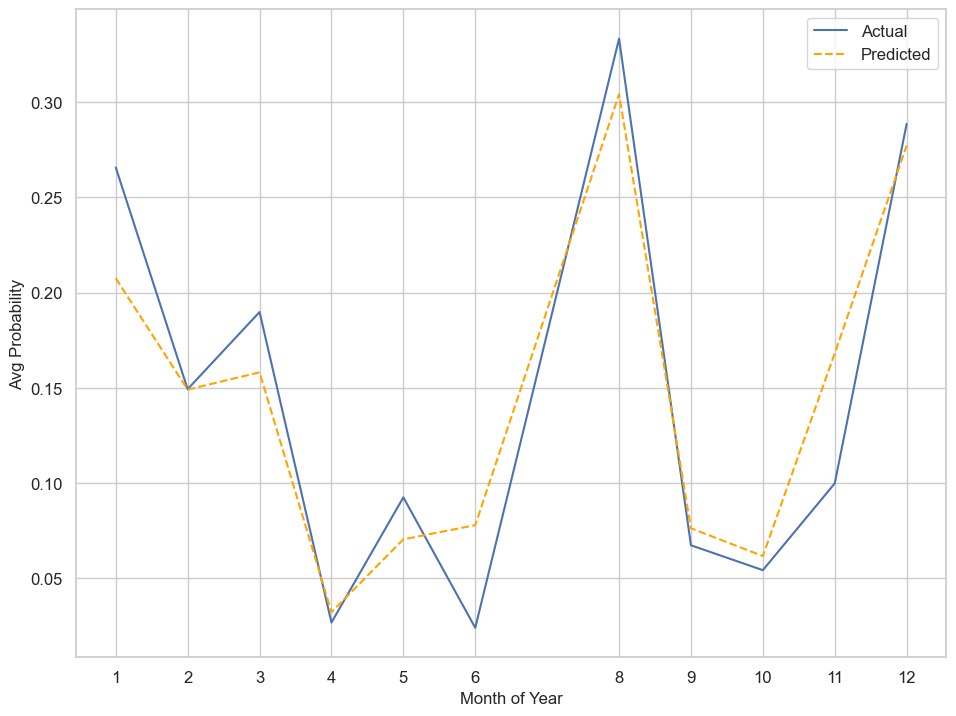 |
| Lake Zoar | 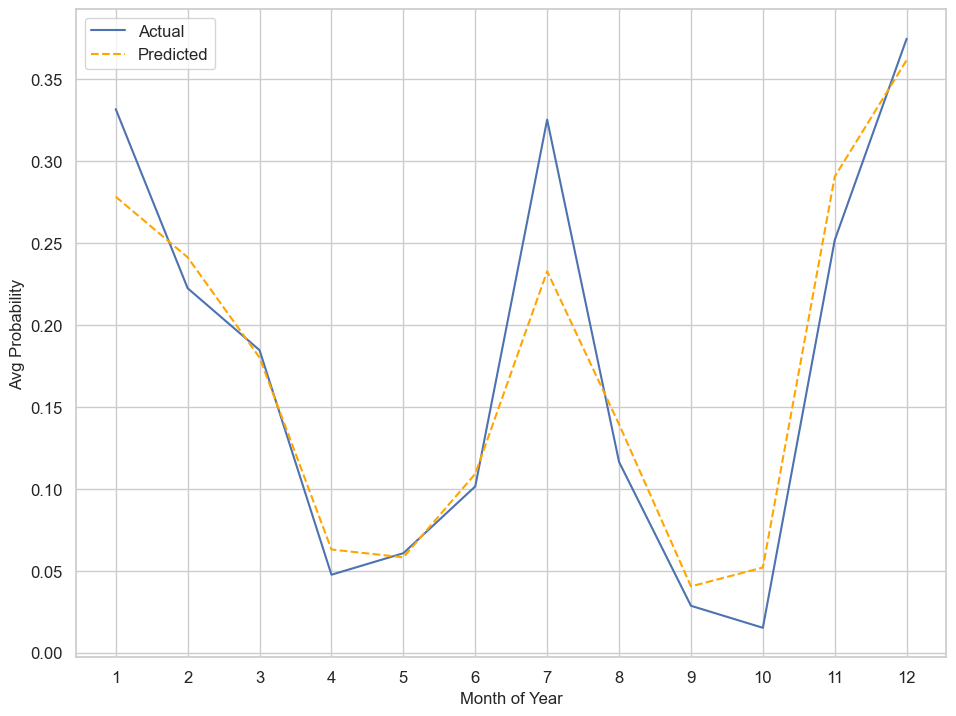 |

Modeled vs Actual probability of elevated PM2.5

The following describes all data and software files and provides a guide for users.

**Contents**:

| **Site** | **File** | **Purpose** | **Use** |
| --- | --- | --- | --- |
| All | DEEP modeling data 2021-1H2022 formatted.csv | Waterbury FEM Monitor data | SQL input (main table) |
| All | Towantic NG Consumption Data Formatted.csv | Natural Gas consumption data for Towantic Energy Ctr | SQL input |
| All | WB_precip formatted.csv | Waterbury precipitation data | SQL input |
| All | NG history East query.csv | Eastern U.S. Natural Gas consumption data | SQL input |
| All | EPA CAMPD Modeling Data.csv | EPA Clean Air Markets Program Data for CPV Towantic Energy Center | SQL input |
| All | OA_Oxford.csv | Oxford “Algonquin” compressor station data | SQL input |
| All | Oxford NOAA dedupe.csv | Waterbury-Oxford Airport NOAA meteorological data | SQL input |
| Gunntown | Purple_gunntown_202101-202206.csv | Purple Air sensor data | SQL input |
| Gunntown | Gunntown SQL.txt | SQL code for Gunntown file processing, in text format | SQL code |
| Gunntown | WB-GT outer join 2021-1H2022 v10.csv (Excel is preferred in Python) | Result of outer join of input files | SQL output / Python input |
| Long Meadow | Purple_longmeadow_202101-202206.csv | Purple Air sensor data | SQL input |
| Long Meadow | Long Meadow SQL.txt | SQL code for Long Meadow file processing, in text format | SQL code |
| Long Meadow | WB-LM outer join 2021-1H2022 v10.csv (Excel is preferred in Python) | Result of outer join of input files | SQL output / Python input |
| Lake Zoar | Purple_woti_202101-202206.csv | Purple Air sensor data | SQL input |
| Lake Zoar | Lake Zoar SQL.txt | SQL code for Lake Zoar file processing, in text format | SQL code |
| Lake Zoar | WB-LZ outer join 2021-1H2022 v10.csv (Excel is preferred in Python) | Result of outer join of input files | SQL output / Python input |
| All | AQ Analytics 10March2023 - Heliyon.ipynb | Python Jupyter code for processing SQL output | Python Jupyter code |
| All | Waterbury PurpleAir Calibration Database.zip | Zipped file of MS Access database; can be used for all data processing | MS Access db |

**Processing Steps**:

1. Locate and download all files from <https://github.com/TKKeyes/HELIYON-D-23-18430.git>
2. Save all files to a suitable computer location in preparation for running SQL
3. For each site (Gunntown, Long Meadow, Lake Zoar), run respective SQL code on input files as noted in the Contents section above
4. Compare resulting SQL output files for each site with “SQL output / Python input” files provided
5. Convert resulting SQL output files from CSV to XLSX if needed (Python works better with *.xlsx)
6. Launch Python/Jupyter (download instructions at <https://www.anaconda.com/download/> - both Jupyter and Spyder interfaces are provided; code provided is Jupyter notebook)
7. Python/Jupyter code is run for each site separately; “uncomment” the appropriate line of code in the section, “Data Reading and Cleaning / Load the dataset”; make sure the files created in step 4) are located in a directory path recognized by Python (i.e., change the file path in the code)
8. Run each section of code sequentially to obtain results for exploratory data analysis, local calibration regressions and local explanatory regressions

If MS Access database can be used –

1. Open database and run the following queries:
   1. WB-GT outer join 2021-1H2022 – NEW
   2. WB-LM outer join 2021-1H2022 – NEW
   3. WB-LZ outer join 2021-1H2022 – NEW
2. For each query run, save the results to CSV or XLSX with file names conforming to files in the Content section above for “SQL output / Python input”

1. 1 Corresponding author. E-mail address: [Tim.Keyes@EGBANA.com](mailto:Tim.Keyes@EGBANA.com) (T. Keyes). [↑](#footnote-ref-1)
2. 2 Tim Keyes was a paid consultant, commissioned by Sierra Club Connecticut for this work. [↑](#footnote-ref-2)
